# Optimised modular anti‐FLAG CAR T cells for solid tumor therapy

**DOI:** 10.1002/cti2.70046

**Published:** 2025-08-21

**Authors:** Xiaomeng Zhang, Rachel Xu, Dmitry Zorin, Evan G Pappas, Jiawei Tang, Yuchen Bai, Vicky M Qin, Bianca von Scheidt, Ruihong Huang, Weronika Kulakowska, Charbel Darido, Phillip K Darcy, Michael H Kershaw, Clare Y Slaney

**Affiliations:** ^1^ Cancer Immunology Program Peter MacCallum Cancer Centre Melbourne VIC Australia; ^2^ Sir Peter MacCallum Department of Oncology The University of Melbourne Parkville VIC Australia; ^3^ Prostate Cancer Research Group, Cancer Program Monash Biomedicine Discovery Institute, Monash University Clayton VIC Australia

**Keywords:** chimeric antigen receptor, FLAG, HER2, solid cancers, tonic signalling

## Abstract

**Objectives:**

Chimeric antigen receptor (CAR) T cell therapies have transformed the treatment of B cell malignancies and show promise in other diseases, including autoimmune disorders and cardiac injury. However, broader application, particularly in solid tumours, is limited by challenges such as antigen escape and tumour heterogeneity. This study aimed to develop an anti‐FLAG CAR capable of engaging FLAG‐tagged secondary reagents, providing a flexible and adaptable targeting strategy.

**Methods:**

We engineered a humanised anti‐FLAG CAR to engage FLAG‐tagged secondary reagents. The initial construct exhibited tonic signalling, which was addressed through structural optimisation. Therapeutic efficacy was assessed in solid tumour mouse models expressing either FLAG or a FLAG‐tagged secondary targeting reagent.

**Results:**

The initial anti‐FLAG CAR showed functional activity but exhibited tonic signalling and exhaustion, limiting its therapeutic utility. Structural optimisation significantly reduced exhaustion and improved T cell persistence and functionality. The optimised CAR T cells effectively inhibited tumour growth in models using either FLAG‐ engineered tumour cells or a FLAG‐tagged secondary targeting reagent.

**Conclusion:**

Our findings underscore the importance of CAR design in minimising exhaustion and enhancing therapeutic efficacy. This work supports a modular CAR T cell platform with the potential to overcome tumour antigen heterogeneity and immune evasion in solid cancers.

## Graphical abstract

This study presents a modular anti‐FLAG CAR T‐cell system that enables flexible tumor targeting through FLAG‐tagged reagents. Structural optimisation of the CAR reduced tonic signalling and glycolytic metabolism, mitigating T‐cell exhaustion.

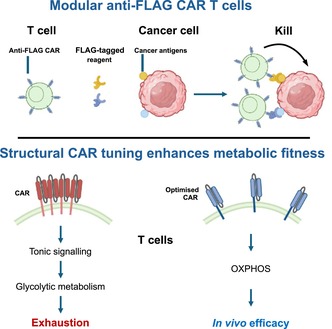

## Introduction

Chimeric antigen receptor (CAR) T‐cell therapies have revolutionised the treatment of B‐cell haematological cancers and are regarded as one of the most potent advancements in cancer treatment.^1^, ^2^ In recent years, CAR T cells have also demonstrated potential in other diseases, such as autoimmune diseases,^3^ infectious diseases^4^ and cardiac injury,^5^ underscoring their broad therapeutic applications.

However, the therapeutic efficacy of CAR T cells in cancer treatment remains inconsistent across cancer types and a significant proportion of patients either exhibit primary resistance or experience disease relapse because of antigen escape—a mechanism whereby cancer cells downregulate or entirely lose target antigen expression.^6^ Compelling evidence suggests that these antigen‐escape patterns are equally pervasive in solid tumors.^7^ Antigen heterogeneity, the diverse and variable expression of tumor antigens, further exacerbates therapeutic challenges.^2^, ^7^

There is a critical need for CAR T‐cell strategies that enable flexible targeting of diverse antigens. This adaptability is essential to address tumor heterogeneity and prevent antigen escape. In this study, we designed a novel humanised anti‐FLAG CAR to function in combination with FLAG‐tagged secondary reagents that specifically bind tumor‐associated antigens. Although the initial anti‐FLAG CAR demonstrated functional activity, *in vitro* culture revealed premature T‐cell exhaustion critically limiting its translational potential. To tackle this challenge, we implemented a structural redesign of the CAR architecture that mitigated tonic signalling, restored physiological signalling and rebalanced metabolic flux towards oxidative phosphorylation. The engineered anti‐FLAG CAR T cells demonstrated robust expansion, exhibited potent antigen‐specific cytotoxicity and secreted elevated IFN‐γ when co‐cultured with target cancer cells.

In NSG mice bearing orthotopic FLAG‐expressing MDA‐MB‐468 breast tumors, the optimised anti‐FLAG CAR T cells achieved complete tumor eradication. These CAR T cells also demonstrated potent efficacy when used in combination with a tumor‐targeting protein bearing a FLAG‐tag for CAR engagement. This modular approach offers a promising strategy for flexible targeting of diverse antigens by using FLAG‐tagged antibodies to bridge anti‐FLAG CAR T cells to tumor cells, with the FLAG epitope serving as a universal docking site to allow rapid antigen switching.

## Results

**Figure 1:**
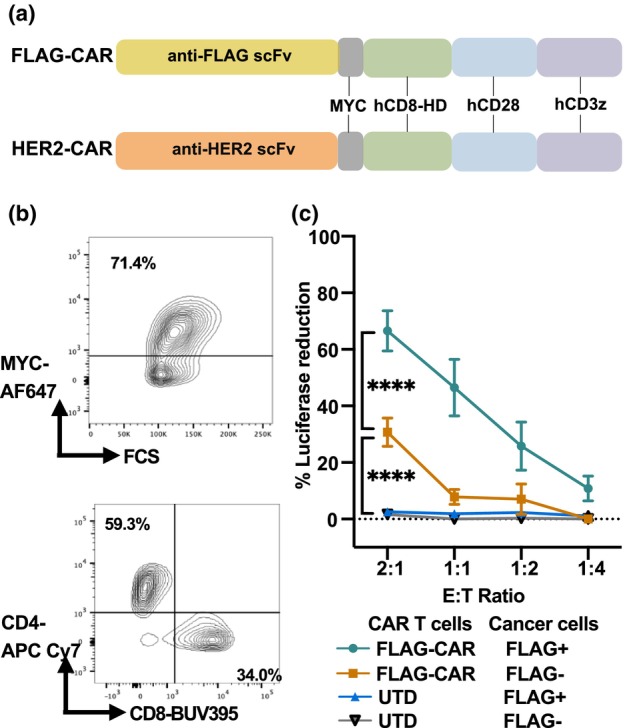
Development of a FLAG‐CAR. **(a)** Diagram illustrating the vector designs for FLAG‐CAR and HER2‐CAR. **(b)** Representative transduction efficiency of chimeric antigen receptor (CAR) T cells and the CD4:CD8 ratio at MOI = 2. **(c)** Cytotoxicity of untransduced (UTD) or FLAG‐CAR T cells against luciferase‐expressing MDA‐MB‐468 (FLAG^−^ or FLAG^+^) cells. Experiments were repeated independently at least three times with consistent results, each performed in triplicate. Representative data with technical replicates were shown as mean ± SEM. Statistical significance: ****P ≤ 0.0001.

Here, we developed an anti‐FLAG CAR (referred to as FLAG‐CAR) using a humanised murine single‐chain variable fragment (scFv) sequence^8^ (Figure 1a). This second‐generation CAR incorporates a human CD28 costimulatory domain and a CD3ζ—chain intracellular signalling domain, connected to the scFv via a human CD8 hinge. To enable visualisation, the construct includes a MYC‐tag and a GFP reporter. The entire construct is driven by a human eukaryotic translation elongation factor 1 alpha (EF‐1α) promoter. As an experimental control, we used an anti‐HER2 CAR (HER2‐CAR) with an identical design, differing only in the scFv, which specifically targets the human HER2 antigen. The selection of HER2‐CAR as a control is because of the fact that HER2‐CAR is clinically validated with a proven safety profile in human trials,^9^, ^10^ and an experimentally optimised system with established protocols has been established in our laboratory's CAR T‐cell research pipeline.^11^, ^12^, ^13^

The FLAG‐CAR lentiviral vector successfully transduced activated T cells derived from human peripheral blood mononuclear cells (PBMC), achieving a robust transduction efficiency consistently exceeding 70% at a multiplicity of infection (MOI) of 2:1 and a balanced CD4:CD8 ratio (Figure 1b). The resulting FLAG‐CAR T cells effectively exert cytotoxicity against cancer cells in an antigen‐specific manner in standard *in vitro* cytotoxicity assays (Figure 1c).

**Figure 2:**
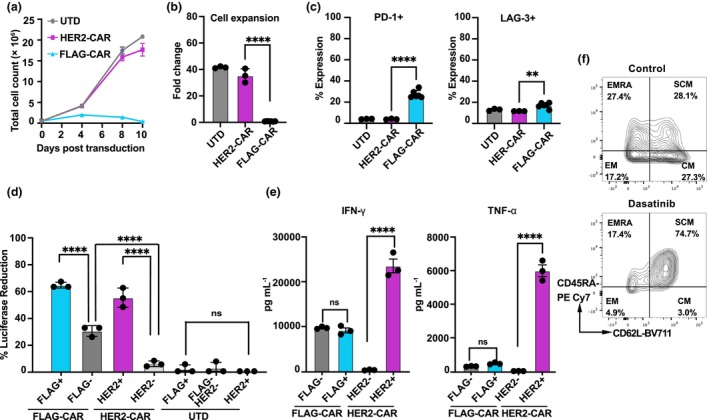
FLAG‐CAR T cells exhibit an exhausted‐like phenotype. **(a)** Growth curves and **(b)** Fold expansion of FLAG‐ and HER2‐CAR T cells over 10 days post‐transduction. **(c)** Percentage of PD‐1 and LAG‐3 expression of anti‐HER2 chimeric antigen receptor (CAR), anti‐FLAG CAR and UTD T cells. **(d)** Cytotoxic activity and **(e)** IFN‐γ and TNF‐α secretion by FLAG‐CAR, HER2‐CAR T cells and UTD T cells. **(f)** Memory phenotypes of FLAG‐CAR T cells without treatment or with the addition of dasatinib at 100 nM from Days 8–15 in culture. Experiments were performed independently at least twice with similar results, each with three to five replicates. Representative data are shown as mean ± SEM with three to five technical replicates. Statistical significance: ** *P* ≤ 0.01, **** *P* ≤ 0.0001.

Although the FLAG‐CAR T cells exhibited overall good functionality, we consistently observed high background killing at high Effector: Target (E:T) ratios *in vitro* (Figure 1c), and they also failed to expand effectively *in vitro*, unlike HER2‐CAR T cells (Figure 2a and b). This limitation was not because of the MOI, as evidenced by preserved expansion kinetics across MOI ratios ranging from 5:1 to 1:1 (Supplementary figure 1). The FLAG‐CAR T cells also exhibited elevated exhaustion markers, including PD‐1 and LAG‐3 (Figure 2c). In addition, FLAG‐CAR T cells demonstrated higher background cytotoxicity (Figure 2d) and lower antigen‐specific secretion of the pro‐inflammatory cytokines IFN‐γ and TNF‐α (Figure 2e). The lack of expansion, increased expression of exhaustion markers and decreased cytokine secretion upon activation have been frequently described as cell exhaustion and attributed to tonic signalling,^14^, ^15^, ^16^ where the exhaustion‐like cell phenotype is induced by antigen‐independent clustering of CARs on T cells.

To confirm the FLAG‐CAR T cells were experiencing tonic signalling, we treated the FLAG‐CAR T cells with dasatinib, a tyrosine kinase inhibitor known to rescue CAR T cells from tonic signalling.^17^ Dasatinib functions by inhibiting downstream signalling pathways of T‐cell activation, thereby preventing continuous activation that can lead to dysfunction. Following incubation with dasatinib for 7 days, the phenotype of the FLAG‐CAR T cells was rescued (Figure 2f) and cell growth restored. These findings further provide evidence that these cells undergo tonic signalling and are progressing to functional exhaustion.

**Figure 3:**
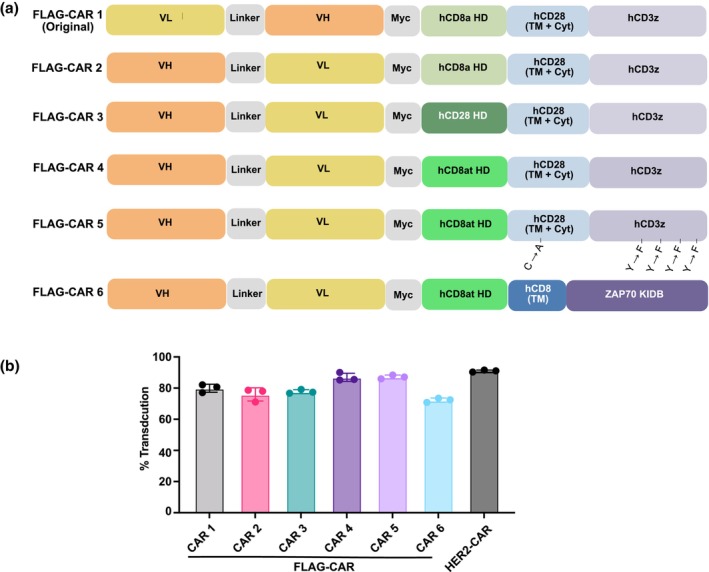
Designs of new FLAG‐CARs. **(a)** Diagram of the new FLAG‐CAR designs. CD8αt: truncated human CD8α hinge sequence. **(b)** Chimeric antigen receptor (CAR) T‐cell transduction efficiency. Experiments were performed independently at least three times with consistent results. Data are represented as mean ± SEM from technical triplicates.

To address the limitations of the FLAG‐CAR T‐cell functions, which were impaired by tonic signalling, we modified the CAR plasmid structure and generated new constructs, as illustrated in Figure 3a. The original design, designated as CAR 1, served as the baseline for comparison. While the scFv region is known to play a pivotal role in altering the tonic‐signalling phenotype,^14^ our options for modification were constrained by the limited availability of scFv sequences. We used a recently developed artificial intelligence (AI)‐based calculator, CAR‐Toner,^16^ to assess the surface electrostatic properties of our CAR constructs by calculating the positively charged patches (PCPs) score. This score quantifies the extent of positively charged regions on the protein surface and serves as a proxy for electrostatic charge, which has been associated with CAR tonic signalling.^14^ Interestingly, our FLAG‐CAR was not predicted to exhibit significantly greater tonic signalling than our HER2‐CAR (FLAG‐CAR score = 54; HER2‐CAR score = 49), suggesting that FLAG‐CAR is not predicted to exhibit significantly higher tonic signalling based on electrostatic surface charge alone. Therefore, we decided to retain the original humanised scFv sequence and implement changes in other parts of the FLAG‐CAR.

Notably, HER2‐CAR T cells, which did not exhibit an exhaustion‐like phenotype, utilised a VH‐VL scFv configuration, whereas the relatively exhausted FLAG‐CAR employed a VL‐VH configuration. Thus, we redesigned the FLAG‐CARs to adopt the VH‐VL configuration. In CAR 2, we modified only the scFv structure by swapping the VH and VL chains while retaining the rest of the original design.

The current food and drug administration (FDA)‐approved CAR T‐cell products incorporate CD8α, CD28 and IgG4 hinge domains.^18^ Numerous studies have demonstrated that the hinge domain, which connects the scFv to the CAR structure and provides flexibility for antigen access on cancer cells, significantly influences CAR T‐cell performance.^18^ Shorter hinge regions have been associated with improved CAR T‐cell efficacy, particularly for targeting membrane‐distal antigens.^19^ To optimise the CAR design, we first modified the hinge. In our original design (CAR 1), we used a human CD8α hinge. One modification involved replacing the CD8α hinge with a human CD28 hinge, which has been reported to enhance CD19‐CAR T‐cell function (CAR 3).^20^ Another approach used a truncated human CD8α hinge sequence (CD8αt), reported to reduce background non‐antigen‐related activity (CAR 4–CAR 6, Supplementary figure 2).^21^ In CAR 5, we also replaced the cysteine (C) residue in the CD28 transmembrane (TM) domain with an alanine (A), a mutation previously reported to reduce tonic signalling.^21^ Additionally, in the immunoreceptor tyrosine‐based activating motifs (ITAMs), we substituted four tyrosines (Y) in ITAMs 2 and 3 with phenylalanines (F). This approach, aimed at reducing phosphorylation and basal CAR T‐cell signalling, was shown to enhance proliferation in response to antigen stimulation and improve CAR T‐cell function *in vivo*.^22^ In CAR 6, we incorporated findings from previous studies, replacing the traditional CD28 and CD3ζ domains with a ZAP70 containing the kinase domain and interdomain B (ZAP70^KIDB^). This design was found to significantly reduce tonic signalling by bypassing upstream CD3ζ and LCK signalling, relying solely on the kinase domain's activity to engage downstream signalling molecules.^21^

**Figure 4:**
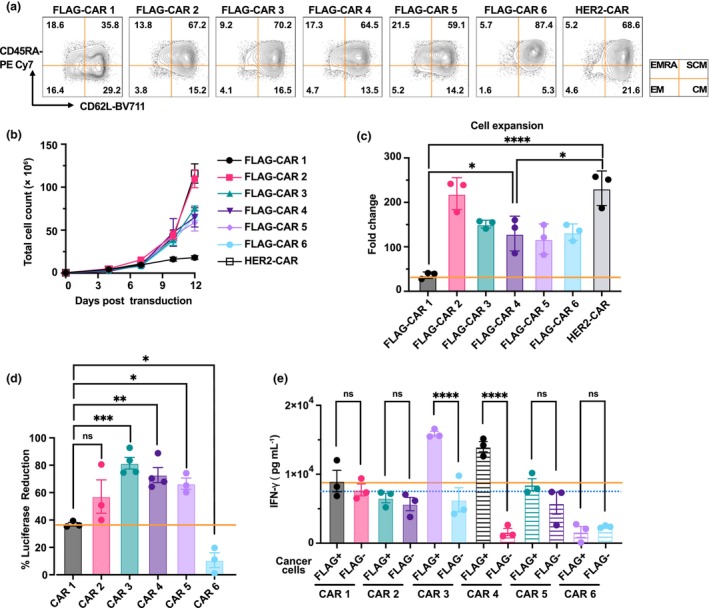
Characterisation of the optimised FLAG‐CARs. **(a)** Phenotypic analysis of new FLAG‐CAR T cells, gated on CD3^+^CAR^+^ cells. **(b, c)** *In vitro* expansion of chimeric antigen receptor (CAR) T cells after CAR transduction. **(d)** Specific cytotoxic activity of CAR T cells against target cells. **(e)** IFN‐γ secretion by CAR T cells co‐incubated with MDA‐MB‐468 (FLAG^−^ or FLAG^+^) cells. Experiments were carried out independently at least twice with consistent results, each with technical triplicates. Data are presented with technical triplicates shown as mean ± SEM, except for Figure 4d (biological replicates, *n* = 3 or 4). Statistical significance: * *P* ≤ 0.05, ** *P* ≤ 0.01, *** *P* ≤ 0.001, **** *P* ≤ 0.0001.

All six CAR constructs were successfully packaged into lentiviral vectors, achieving transduction rates exceeding 70% at an MOI of 2:1 in human T cells (Figure 3b). Excitingly, all the new CARs (CAR 2–CAR 6) exhibited improved phenotypes compared to the original FLAG‐CAR (CAR 1). While only ~30% of CAR1‐transduced cells displayed a T_SCM_‐like phenotype, > 50% of cells transduced with CAR 2–CAR 5 and HER2‐CAR were T_SCM_‐like (Figure 4a). The new CARs showed significantly better expansion *in vitro*. Similar to the non‐exhausted HER2‐CAR, the five new FLAG‐CARs expanded ~150–200 fold within 2 weeks. In contrast, CAR 1 failed to expand and eventually led to a collapse of the cell culture (Figures 4b and c and 2a). *In vitro* assays further demonstrated the superior functionality of FLAG‐CAR 3–5. These three new CAR T cells showed enhanced specific killing of FLAG^+^ cancer cells compared to CAR 1 (Figure 4d). Additionally, CAR 3 and CAR 4 T cells secreted significantly higher levels of IFN‐γ when co‐cultured with FLAG^+^ cancer cells, further confirming their improved specificity and reduced exhaustion (Figure 4e). Although CAR 2 and CAR 6 demonstrated a strong expansion rate and favorable memory phenotype *in vitro*, the new design did not improve the functionality of these two CARs. CAR 2 did not show any significantly enhanced effect on specific killing compared to CAR 1 (Figure 4d). It maintained a high background IFN‐γ secretion level while exhibiting limited ability to secrete IFN‐γ when incubated with FLAG^+^ cancer cells (Figure 4e). This suggests ineffective activation coupled with off‐target activity. CAR 6 completely lacked both antigen‐specific cytotoxicity and cytokine response (Figure 4d and e), indicating a non‐functional receptor configuration.

**Figure 5:**
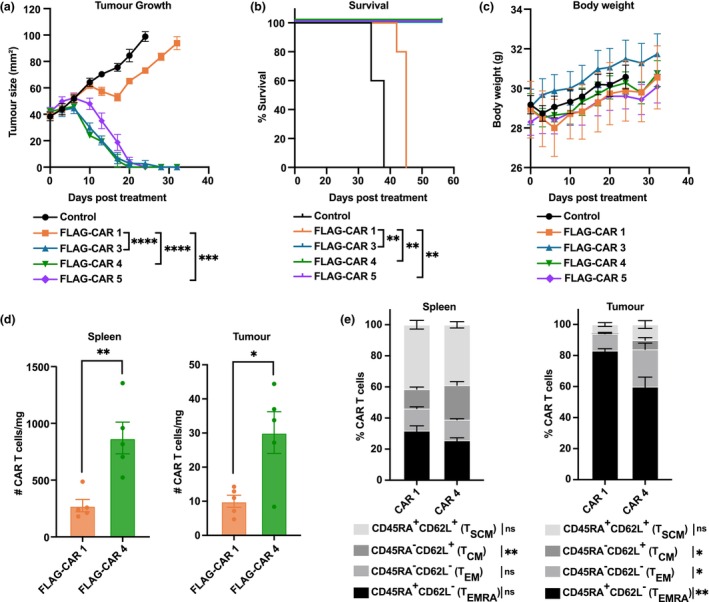
*In vivo* functional comparison of the anti‐FLAG chimeric antigen receptor (CAR) T cells. **(a)** MDA‐MB‐468‐FLAG tumor growth in NSG mice. After 15 days of mammary gland tumor engraftment, NSG mice received 0.5 Gy total body irradiation and treatment with 2 × 10^6^ CAR T cells (FLAG‐CAR 4, *n* = 4; other groups, *n* = 5). **(b)** Survival of mice. Endpoint is defined as tumor size > 100 mm^2^. **(c)** Body weight. **(d)** NSG mice bearing MDA‐MB‐468‐FLAG mammary gland tumors received 5 × 10^6^ FLAG‐CAR 1 or FLAG‐CAR 4 T‐cell treatment after 28 days of tumor engraftment. Data are shown as normalised CAR T‐cell numbers in spleens and tumors, seven days after CAR T‐cell treatment (*n* = 5). **(e)** Phenotypes of CAR T cells in **(d)**. Experiments have been independently performed at least twice. Data are presented as mean ± SEM from one experiment. Statistical significance: * *P* ≤ 0.05, ** *P* ≤ 0.01, *** *P* ≤ 0.001, **** *P* ≤ 0.0001.

We next evaluated the anti‐tumor effects of the most promising CARs, CAR 3–CAR 5, using a FLAG^+^ solid tumor model. In this model, MDA‐MB‐468 human breast cancer cells were orthotopically engrafted into the mammary glands of immunocompromised NSG mice. To simulate a solid tumor setting, where a FLAG‐tagged scFv binds the tumor antigen on the tumor cell surface, the MDA‐MB‐468 cells were transduced with a non‐functional scFv containing a C‐terminal FLAG peptide and a transmembrane domain (Supplementary figure 3). This construct, which lacks antigen‐binding capacity, anchors the FLAG peptide to the tumor cell surface and is hereafter referred to as MDA‐MB‐468‐FLAG. Once established, the tumors were treated with FLAG‐CAR T cells. Remarkably, CAR 3–CAR 5 effectively inhibited the growth of orthotopic MDA‐MB‐468‐FLAG tumors in NSG mice, achieving complete eradication of the cancers (Figure 5a) and extended survival (Figure 5b). In contrast, the original CAR 1 that exhibited tonic signalling showed only a marginal effect on MDA‐MB‐468‐FLAG tumor progression (Figure 5a). There was no overt toxicity observed in any treatment groups (Figure 5c).

Among the three effective FLAG‐CARs (CAR 3, CAR 4 and CAR 5), we selected CAR 4 for further investigation because of its superior *in vitro* and *in vivo* performance. Although CAR 3 demonstrated strong *in vivo* efficacy and specific killing, it consistently exhibited high background IFN‐γ secretion (Figure 4e). CAR 5 lacked IFN‐γ secretion upon co‐culture with target cancer cells (Figure 4e). As shown in Supplementary figure 4, no significant differences were observed among FLAG‐CAR 1, FLAG‐CAR 4 and HER2‐CAR T cells in terms of transduction efficiency, CAR surface expression or CD4^+^/CD8^+^ T‐cell composition across four individual donors. These results indicate that the observed functional differences between the CAR constructs are unlikely to be as a result of variability in transduction levels or T‐cell subset distribution. Considering these factors, we prioritised CAR4 for further study to understand the mechanisms underlying its superior performance and advance its development towards clinical translation.

Flow cytometric analysis revealed that 7 days post‐treatment, CAR 4‐treated mice had significantly more CAR T cells in both the spleens and tumors of the MDA‐MB‐468‐FLAG tumor‐bearing mice compared to CAR 1‐treated mice (Figure 5d). In addition, CAR 4 T cells in the spleens and tumors had a higher proportion of cells with a T_CM_‐like phenotype (Figure 5e), which are typically associated with superior *in vivo* efficacy, expansion and persistence.^1^, ^23^ In contrast, tumors from CAR 1‐treated mice contained significantly more cells with T_EM_ and T_EMRA_ phenotypes. These cells are considered fully differentiated, have a short lifespan and limited *in vivo* expansion capacity.^1^, ^23^

**Figure 6:**
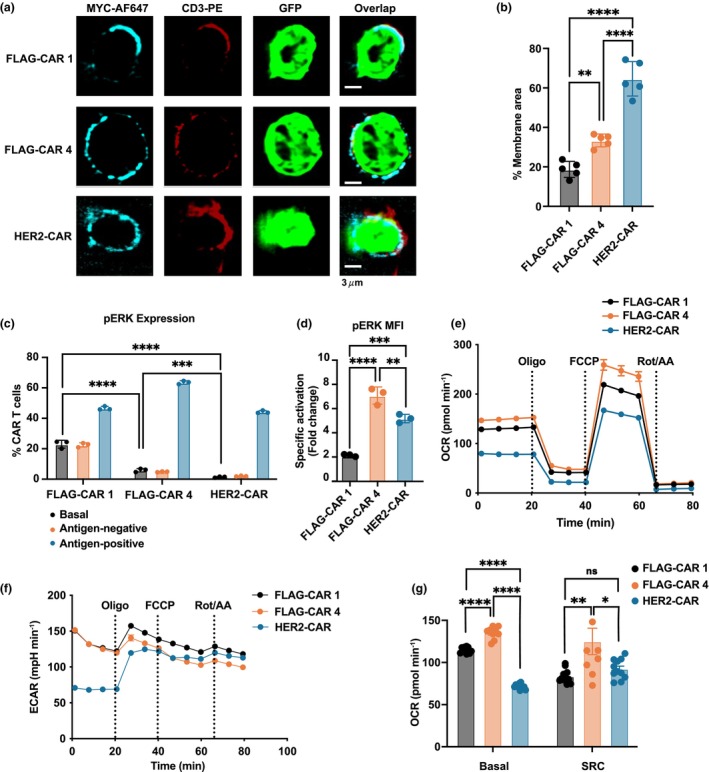
Enhanced signalling and metabolism of the optimised CAR4. **(a)** Lattice Lightsheet microscopy images showing chimeric antigen receptor (CAR) (MYC), CD3 and GFP expression on CAR T cells in the absence of antigen stimulation. Scale bars, 3 μm. **(b)** Percentage of membrane area expressing CAR. **(c)** pERK signalling in CAR T cells at a resting state (basal), and after incubation with antigen‐positive (FLAG^+^ or HER2^+^) or antigen‐negative (FLAG^−^ HER2^−^) cancer cells at a ratio of 1:1 for 30 min. **(d)** Fold change in pERK mean fluorescence intensity (MFI) over 30 min of incubation with antigen‐positive cells, normalised to resting state. **(e)** Oxygen consumption rate (OCR) and **(f)** extracellular acidification rate (ECAR) curves from Seahorse assay. Oligo, oligomycin; FCCP, Carbonyl cyanide‐p‐trifluoromethoxyphenylhydrazone; rot/AA, rotenone and antimycin A. **(g)** Basal and spare respiratory capacity of FLAG‐CAR 1, FLAG‐CAR 4 and HER2‐CAR T cells. Experiments were performed independently at least twice with consistent results. Data are presented with technical replicates shown as mean ± SEM. Statistical significance: * *P* ≤ 0.05, ** *P* ≤ 0.01, *** *P* ≤ 0.001, **** *P* ≤ 0.0001.

Confocal microscopy revealed that CAR 4 exhibited normal clustering on the T‐cell surface with a relatively even distribution, closely resembling the HER2‐CAR (Figure 6a and b). In contrast, CAR 1 displayed a punctate phenotype, characteristic of high tonic‐signalling CARs reported in previous studies.^14^, ^15^

Additionally, ERK phosphorylation, a marker of MAPK‐driven tonic signalling,^14^ is significantly lower in CAR 4 cells than in CAR 1, indicating reduced tonic signalling in CAR 4 (Figure 6c). Upon antigen stimulation, CAR 4 cells exhibit approximately 3.5‐fold higher pERK expression than CAR 1 cells, suggesting enhanced antigen‐specific activation. Notably, this activation was even greater than that observed in HER2‐CAR cells (Figure 6d). This pattern suggests that CAR 4 maintains a lower basal activation state, while retaining the capacity for robust ERK activation upon stimulation. Given the role of the MAPK pathway in metabolic regulation, this dynamic ERK response may contribute to enhanced mitochondrial function, supporting a metabolically fitter phenotype in CAR 4 compared to CAR 1 T cells.

We subsequently performed a Seahorse assay to compare the metabolism of CAR 4 and CAR 1, using HER2‐CAR as the experimental control. As HER2‐CAR differs from FLAG‐CARs in multiple domains, including scFv and hinge, it was used solely as a positive control rather than for direct comparison. Interestingly, the results revealed that the HER2‐CAR differs significantly from the FLAG‐CARs, exhibiting lower basal and maximal respiration rates, as well as a distinct extracellular acidification rate (ECAR) (Figure 6e and f), highlighting the fundamental differences between HER2‐CAR and FLAG‐CAR T cells. Between FLAG‐CARs, CAR 4 outperformed CAR 1, demonstrating higher oxidative phosphorylation (OXPHOS) reflecting a higher mitochondrial respiration, as reflected by an increased basal oxygen consumption rate (OCR) and a higher spare respiratory capacity (SRC) (Figure 6g). Additionally, CAR 4 showed reduced glycolysis, as indicated by a lower ECAR. The reduced ECAR and enhanced OXPHOS indicate that CAR 4 T cells have a shift towards mitochondrial respiration for oxidative metabolism and rely less on glycolysis for energy production.^24^, ^25^ These data indicate that CAR 4 T cells are adopting a more efficient, oxidative metabolic state, which is often associated with enhanced mitochondrial function and overall cellular fitness. Together, our data indicate a more metabolically fit phenotype of CAR 4 than of CAR 1 T cells. The link between ERK activation and mitochondrial fitness further supports the notion that CAR 4 and anti‐HER2 CAR T cells maintain a less differentiated and energetically efficient state.

**Figure 7:**
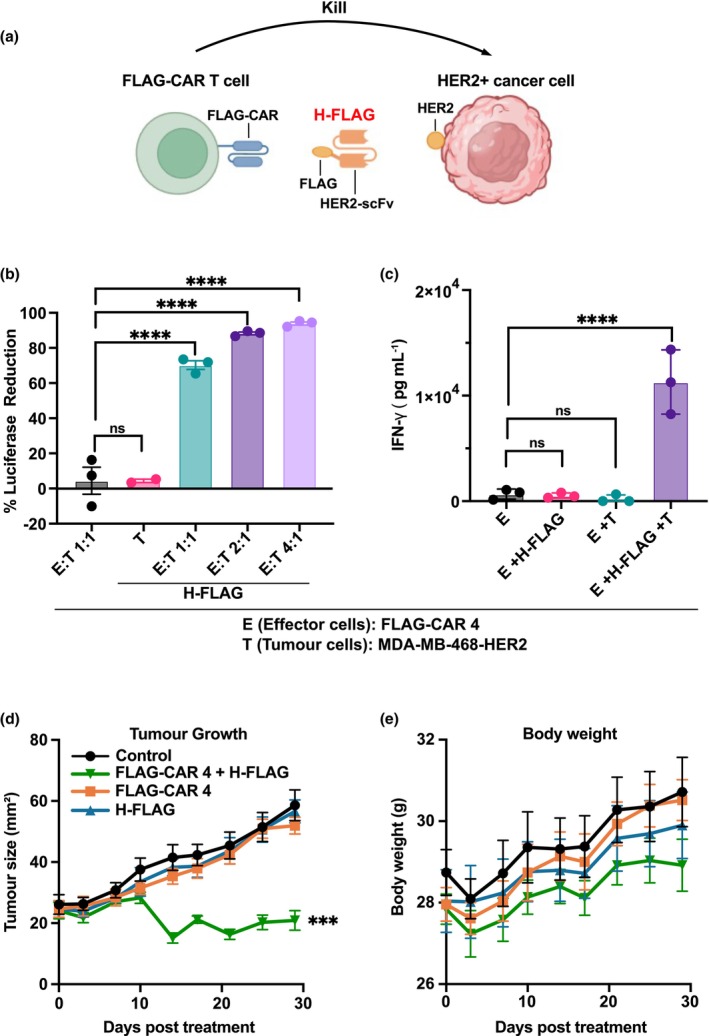
H‐FLAG enables tumor suppression by FLAG‐CAR 4 T cells. **(a)** Schematic of the H‐FLAG protein used to redirect FLAG‐CAR 4 T cells towards HER2^+^ tumor cells (generated with Biorender). **(b)** *In vitro* cytotoxicity of FLAG‐CAR 4 T cells against MDA‐MB‐468‐HER2‐luc cells in the presence or absence of H‐FLAG, measured by luciferase release. **(c)** IFN‐γ secretion by FLAG‐CAR 4 T cells co‐cultured with MDA‐MB‐468‐HER2 target cells, with or without H‐FLAG. **(d)** Tumor growth curves of NSG mice bearing orthotopic MDA‐MB‐468‐HER2 tumors treated with FLAG‐CAR 4 T cells, H‐FLAG, both or control. Tumor volume was measured over time. **(e)** Body weight. Experiments were performed independently at least twice with consistent results. Data are presented as mean ± SEM. CAR, chimeric antigen receptor. Statistical significance: *** *P* ≤ 0.001, **** *P* ≤ 0.0001.

To evaluate the therapeutic potential of FLAG‐CAR 4 T cells in combination with a tumor‐targeting FLAG‐tagged reagent, we engineered a recombinant protein consisting of an anti‐HER2 scFv fused to a FLAG tag as the core targeting element (Figure 7a). The construct also includes additional components, such as an Fc region and flexible linkers, to enhance structural flexibility, stability and serum half‐life. This molecule is hereafter referred to as H‐Flag (HER2‐scFv‐FLAG). H‐FLAG enabled robust and specific activation of FLAG‐CAR 4 T cells against HER2^+^ tumor cells, resulting in potent cytotoxicity (Figure 7b) and IFN‐γ secretion (Figure 7c), only when both the HER2‐scFv‐FLAG and tumor antigen were present. Furthermore, in the orthotopic MDA‐MB‐468‐HER2 breast cancer model, FLAG‐CAR 4 T cells alone or the H‐FLAG alone had minimal impact on tumor growth, but their combination led to significant tumor regression (Figure 7d), demonstrating the efficacy of this modular targeting strategy *in vivo*. No overt toxicity was observed in treated mice (Figure 7e), supporting the safety of this approach.

Overall, our data showed that the optimised CAR 4 T cells outperform CAR 1 T cells. The CAR structure itself likely influences intracellular signalling, which in turn shapes the metabolic profile of CAR T cells, ultimately affecting their *in vitro* function and *in vivo* therapeutic performance. Furthermore, the use of an H‐FLAG protein enabled modular redirection of FLAG‐CAR T cells towards HER2^+^ tumors, resulting in effective tumor clearance. These findings highlight the versatility and therapeutic potential of the anti‐FLAG CAR platform in enabling flexible targeting of solid tumors.

## Discussion

CAR T‐cell therapy has shown remarkable success in treating haematological malignancies, but its efficacy in solid tumors remains limited because of major challenges like antigen heterogeneity and antigen loss. Unlike blood cancers, where a single antigen is typically uniformly expressed, solid tumors show high variability in antigen expression both across tumor types and within individual tumors, making target identification difficult and increasing the risk of treatment failure. Additionally, antigen loss is a significant challenge, as tumor cells can downregulate or mutate the targeted antigen to evade immune recognition, a process facilitated by genomic instability and clonal diversity. These factors contribute to treatment resistance, tumor recurrence and poor therapeutic outcomes, underscoring the need for strategies that enable flexible and broad targeting of tumor antigens.

To overcome these limitations, researchers have explored targeting exogenous antigens and using modular CAR systems that allow adaptable antigen recognition. One approach employs a CAR specific to fluorescein isothiocyanate (FITC), used in combination with an intratumorally injected amphiphile conjugated to FITC to treat solid tumors.^26^ This enhances CAR T‐cell infiltration and promotes systemic anti‐tumor immunity, including abscopal effects and immune memory formation. Other universal CAR strategies include the SUPRA (split, universal and programmable) CAR^27^ and convertible CAR.^28^ These systems separate the CAR recognition from antigen specificity, using adaptor molecules to guide CAR T cells to different antigens. For instance, the SUPRA CAR uses a leucine zipper‐based adaptor system, while convertible CAR employs an inert NKG2D domain that binds to ULBP2‐based adaptors fused to tumor‐targeting antibodies. These designs enable controlled and flexible targeting of diverse antigens and allow for rapid reprogramming of CAR specificity.

Building on this concept, the current study developed a CAR targeting the FLAG‐tag, an exogenous peptide widely used in molecular biology. A key advantage of this platform is its compatibility with a broad range of tumor‐targeting antibodies and moieties, which can be readily adapted by appending a short FLAG‐tag. This modular approach not only offers flexibility and rapid antigen retargeting but also reduces the risk of off‐target toxicity, as the FLAG‐tag is not naturally expressed in human tissues. Although a theoretical risk of anti‐FLAG immune responses exists, our previous two clinical trials using a Lewis‐Y CAR incorporating a FLAG‐tag reported no associated toxicity or CAR T‐cell loss.^29^, ^30^

Our initial FLAG‐CAR design showed evidence of tonic signalling, leading to T‐cell exhaustion. To address this, several structural modifications were tested, including domain reordering, hinge shortening and substitution of costimulatory domains.^14^, ^15^, ^16^, ^21^ The optimised CAR, which is CAR 4, featured a truncated version of the hCD8α hinge and showed reduced tonic signalling, improved metabolic fitness and enhanced effector function. CAR4 successfully eliminated FLAG‐expressing breast cancer in the mouse models. Moreover, when used in combination with a FLAG‐tagged HER2‐scFv, CAR 4 induced significant regression of orthotopic HER2^+^ tumors, highlighting the therapeutic potential of this flexible targeting strategy.

In summary, this study establishes the FLAG‐CAR as a flexible and modular platform for CAR T‐cell therapy. By addressing tonic signalling and improving T‐cell function, the optimised FLAG‐CAR demonstrates effective anti‐tumor activity. The combination of FLAG‐tagged tumor‐targeting moieties with FLAG‐CAR offers a potential strategy for adaptable redirection towards diverse tumor‐associated antigens.

## Methods

### CAR T‐cell production

Peripheral blood mononuclear cells were obtained from fresh buffy coats supplied by the Australian Red Cross Blood Service and isolated using a Ficoll density gradient. The PBMCs were then cultured in TexMACS medium (Miltenyi Biotec B.V. & Co. KG, Bergisch Gladbach, Germany) supplemented with recombinant human IL‐7 and IL‐15 (Miltenyi Biotec B.V. & Co. KG) at 12.5 ng mL^−1^ and T‐cell TransAct (Miltenyi Biotec B.V. & Co. KG) at a 1:100 dilution. After 48 h, the activated T cells were transduced with concentrated CAR lentiviral vectors and subsequently maintained in TexMACS medium containing 12.5 ng mL^−1^ of recombinant human IL‐7 and IL‐15.

CAR T constructs were synthesised by Genscript Singapore and cloned to pCDH vector (Addgene, Watertown, USA). The CAR T plasmids were mixed with pMDLg/pRRE, pRSV‐Rev and pMD2.G plasmids (Addgene) and transfected into HEK 293 T cells using Lipofectamine 3000 (Thermo Fisher Scientific, Waltham, USA) according to the manufacturer's protocol. Supernatant containing the CAR lentiviral vectors was harvested, concentrated by ultracentrifugation with Optima XPN‐100 (Beckman Coulter, Brea, USA), titrated and stored at −80°C.

### Cytotoxicity assays

Luciferase‐expressing target tumor cells were plated in a white 96‐well microplate (Revvity, Waltham, USA). CAR T cells were added at various E:T ratios as indicated. Controls included target cells alone and target cells treated with 10% SDS. Following an overnight incubation at 37°C, VivoGlo Luciferin (Promega, Madison, USA) was added at a final concentration of 300 μg mL^−1^. Luminescence was measured using BioTek Cytation 3 (Agilent Technologies, Santa Clara, USA). The percentage of luciferase reduction was calculated as follows:

$$\left(\text{target cell alone luminescence}\;-\;\text{sample luminescence}\right)/\left(\text{target cell alone luminescence}\;-\;\text{target cell in}\;10\%\mathrm{SDS}\;\text{luminescence}\right)\times 100\%.$$

### Cytokine assays

Chimeric antigen receptor T cells were seeded to 96‐well flat‐bottom plates with an equal number of tumor cells in complete RPMI and incubated at 37°C overnight. Supernatant was collected, and cytokines were analysed using the AlphaLISA kits according to the manufacturer's protocol (Revvity, Waltham, USA). Plates were read on the BioTek Cytation 5 (Agilent Technologies, Santa Clara, USA).

### Flow cytometry

Cell surface flow cytometric analysis was performed as previously described.^11^, ^12^, ^13^ For intracellular staining, FLAG‐ or HER2‐CAR T cells were incubated with NALM6‐FLAG or NALM6‐HER2, respectively, at a 1:1 ratio in RPMI containing 0.1% fetal bovine serum (FBS) for 30 min in a 37°C water bath. At the end of incubation, samples were immediately fixed in 1:1 volume of BD Cytofix for 10 min at room temperature, then permeabilised in methanol for 10 min on ice. Samples were stained with anti‐ERK1/2 phospho (T202/Y204) antibody (clone 6B8B69, BioLegend, San Diego, USA) in buffer [phosphate‐buffered saline (PBS) containing 1% FBS and 0.09% sodium azide] for 1 h in the dark on ice. Samples were analysed on BD FACSCanto II, BD LSR II, BD LSRFortessa X‐20, BD FACSymphony A3 or A5 (BD Bioscience, San Diego, USA). Data are analysed and plotted using FlowJo™ v10.10 (BD Bioscience).

### Mouse experiments and cell lines

All mouse experiments were approved by the Peter MacCallum Cancer Centre Animal Experimentation Ethics Committee. NOD.Cg‐Prkdc scid Il2rg tm1Wjl/SzJ mice (NSG) were bred at the Peter MacCallum Cancer Centre (Melbourne, Australia), and mice used for experiments were between 6 and 10 weeks of age. Experiments were conducted using a mixed cohort of male and female mice based on availability.

In the xenograft models, 1 × 10^6^ MDA‐MB‐468‐FLAG cells in 20 μL PBS were injected into the left fourth mammary glands. Tumor areas were measured with a calliper twice per week and calculated as length × width. In the tumor growth and survival study, mice received 0.5 Gy total body irradiation 15 days following tumor injection and on the same day, 2 × 10^6^ CAR T cells were injected intravenously in 200 μL PBS. In the mechanistic study, mice received 0.5 Gy total body irradiation 28 days after tumor injection, followed by intravenous injection of 5 × 10^6^ CAR T cells in 200 μL PBS. Mice were harvested 7 days after CAR T‐cell treatment. Tumors were enzymatically digested at 37°C for 20 min with 1 mg mL^−1^ Collagenase IV (Worthington Biochemical, Lakewood, USA), 100 μg mL^−1^ Hyaluronidase and 75 μg mL^−1^ DNase I (Sigma‐Aldrich, St. Louis, USA) in RPMI‐1640 medium before mechanical dissociation through a 70‐μm cell strainer. Spleens were mechanically dissociated through a 70‐μm cell strainer before red blood cell lysis. Cell suspensions were used for surface staining and analysed by flow cytometry as described above.

To assess the *in vivo* efficacy of the FLAG‐CAR 4 T cells in combination with the H‐FLAG protein (synthesised by GenScript, Nanjing, China), NSG mice (6–8 weeks of age) were orthotopically injected with 1 × 10^6^ MDA‐MB‐468‐HER2 tumor cells into the fourth mammary fat pad. Thirteen days after the tumor injection, mice were randomly assigned into four treatment groups: (1) untreated control, (2) FLAG‐CAR 4 T cells alone, (3) H‐FLAG alone and (4) FLAG‐CAR 4 T cells plus H‐FLAG. Mice received a single intravenous injection of 5 × 10^6^ FLAG‐CAR4 T cells, with or without co‐administration of 200 μg H‐FLAG on treatment days 0, 7 and 18 for a total of 3 doses. Tumor area and body weight were monitored twice per week using digital callipers and a standard formula (length × width). Mice were euthanised when tumors exceeded ethical limits or at the study endpoint.

All cell lines were originally purchased from ATCC and have been authenticated in‐house. Mycoplasma testing is conducted regularly, and only mycoplasma‐negative cell lines are used in our studies. Cancer cells were transduced to express HER2, FLAG or other antigens, such as luciferase, as previously described.^11^, ^12^, ^13^

### Confocal microscopy

Live GFP^+^ CAR‐T cells were incubated with anti‐MYC‐AF647 (clone 9B11; Cell Signaling Technology, Danvers, USA) and anti‐CD3‐PE (clone SK7; BioLegend, San Diego, USA) antibodies for 30 min at room temperature and imaged using a Lattice Lightsheet microscope (Carl Zeiss AG; Oberkochen, Germany). Fluorescence signals were split using a 640‐nm long‐pass dichroic mirror and filtered with a 495–550‐nm bandpass for PE/GFP and a 570–620‐nm bandpass for AF647. Five images for each group were acquired. The membrane areas expressing CAR (MYC‐AF647^+^) were quantified using Imaris software (v10.1; Oxford Instruments, Abingdon, England). Images were pre‐processed to subtract background, and masks were generated for each channel using identical thresholding and voxel minimum parameters across all samples to ensure consistency. Cell surfaces were segmented based on cytoplasmic GFP, and MYC‐AF647 signal was confined to these segmented surfaces. Intensity measurements and spatial distribution analyses were performed using the Statistics and Spots tools, with voxel size calibrated to acquisition settings.

### Seahorse assay

4 × 10^5^/well of CAR T cells were seeded to XFe96 cell culture microplates pre‐coated with CellTak (Corning, New York, USA) in XF base media containing 11 mM glucose, 2 mM L‐glutamine and 1 mM sodium pyruvate. The OCR and ECAR were measured by using the Seahorse XF Pro analyser (Agilent Technologies, Santa Clara, USA) at baseline and after sequential injection of oligomycin (1 μM; Merck Life Science, Bayswater, Australia), FCCP (1.5 μM; Merck Life Science), rotenone (100 nM; Merck Life Science) and antimycin A (1 μM; Merck Life Science).

### Graphical presentation and statistical analysis

Data were analysed and graphed using GraphPad Prism 10 (GraphPad Software, La Jolla, USA) and Microsoft Excel (Microsoft Corporation, Albuquerque, USA). One‐way ANOVA and Tukey's multiple comparisons test or Dunnett's multiple comparisons test were used to compare groups of data. Student's *t*‐test was employed to compare two groups of data. Mouse survival was compared using the Mantel‐Cox test. Significant differences between comparisons were indicated by *P*‐values (* ≤ 0.05, ** ≤ 0.01, *** ≤ 0.001, **** ≤ 0.0001), not significant (ns).

## Author contributions

**Xiaomeng Zhang:** Investigation; methodology; software; data curation; validation; visualization; writing – original draft; writing – review and editing; formal analysis. **Rachel Xu:** Methodology; data curation; investigation; validation; formal analysis; visualization; writing – review and editing. **Dmitry Zorin:** Writing – review and editing; methodology; formal analysis; validation; investigation; data curation. **Evan G Pappas:** Methodology; validation; investigation; visualization; formal analysis; data curation; writing – review and editing. **Jiawei Tang:** Writing – review and editing; methodology; investigation; validation; visualization; formal analysis; data curation. **Yuchen Bai:** Methodology; validation; investigation; visualization; software; formal analysis; data curation; writing – review and editing. **Vicky M Qin:** Methodology; software; data curation; investigation; validation; formal analysis; visualization; writing – review and editing. **Bianca von Scheidt:** Methodology; data curation; investigation; validation; formal analysis; supervision; project administration; visualization; writing – review and editing. **Ruihong Huang:** Methodology; data curation; software; investigation; validation; visualization; writing – review and editing. **Weronika Kulakowska:** Writing – review and editing; methodology; investigation; validation. **Charbel Darido:** Writing – review and editing; supervision; conceptualization. **Phillip K Darcy:** Writing – review and editing; funding acquisition; project administration; resources; supervision; conceptualization. **Michael H Kershaw:** Writing – review and editing; funding acquisition; project administration; resources; supervision; formal analysis; methodology; conceptualization. **Clare Y Slaney:** Conceptualization; investigation; funding acquisition; writing – original draft; writing – review and editing; visualization; validation; methodology; software; formal analysis; project administration; resources; supervision; data curation.

## Conflict of interest

The authors declare no conflict of interest.

## Supporting information


Supplementary figure 1
Supplementary figure 2
Supplementary figure 3
Supplementary figure 4

## Data Availability

The data supporting the findings of this study are available from the corresponding author upon reasonable request.
