# Supplementary material for: Optimised modular anti‐FLAG CAR T cells for solid tumor therapy
Source: Clin Transl Immunology. 2025 Aug 21;14(8):e70046. doi: 10.1002/cti2.70046 (PMC12370372; doi:10.1002/cti2.70046)
Supplement: Supplementary file 1 — Supplementary figure 1 Supplementary figure 2 Supplementary figure 3 Supplementary figure 4 [file CTI2-14-e70046-s001.pdf]

Supplementary figure1

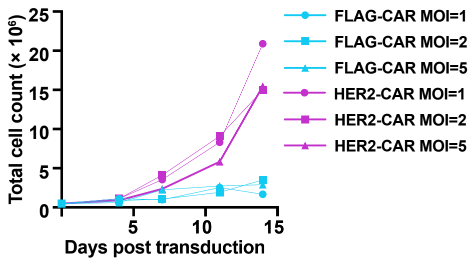

Supplementary figure 1. The growth curve of FLAG-CAR T cells *in vitro*. Growth curves of FLAG-CAR (CAR1) and HER2-CAR T cells transduced with the MOI = 1, 2, 5.

Supplementary figure 2

```
CD8at --------------------TTTPAPRPPTPAPTIASQPLSLRPEACRPAAGGAVHTRGLDFACD 45
CD8a  LSNSIMYFSHFVPVFLPAKPTTTPAPRPPTPAPTIASQPLSLRPEACRPAAGGAVHTRGL----- 60
                               ***********************************
```

Supplementary figure 2. The alignment of the CD8a hinge and CD8at hinge. EMBOSS Needle[31] was used to compare the CD8a hinge and CD8at hinge. Job ID: emboss_needle-I20241227-052703-0228-27753090-p1m.

Reference: Madeira F, Madhusoodanan N, Lee J, Eusebi A, Niewielska A, Tivey ARN *et al*. The EMBL-EBI Job Dispatcher sequence analysis tools framework in 2024. *Nucleic Acids Res* 2024; **52**: W521-W525.

Supplementary figure 3

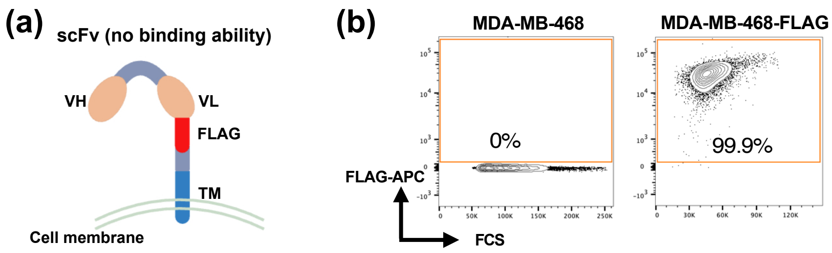

Supplementary figure 3. Generation of MDA-MB-468-FLAG tumour cells. **(a)** Schematic of the membrane-anchored FLAG construct (generated with Biorender). A non-functional scFv lacking antigen-binding ability was fused to a FLAG tag and a transmembrane (TM) domain to enable stable surface expression on tumour cells. **(b)** Flow cytometry analysis showing FLAG surface expression on parental MDA-MB-468 cells (**left**) and MDA-MB-468 cells transduced with the FLAG construct (**right**).

Supplementary figure 4

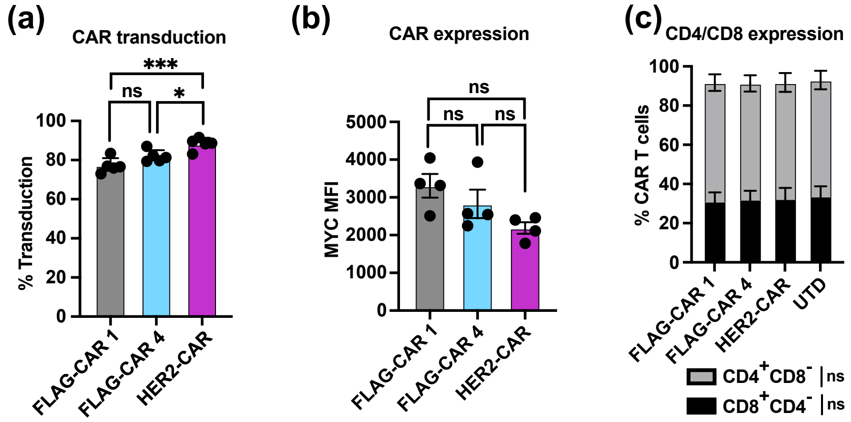

Supplementary figure 4. CAR transduction efficiency, surface expression, and CD4/CD8 distribution across different CAR constructs. (a) Transduction efficiency of FLAG-CAR 1, FLAG-CAR 4, and HER2-CAR T cells, assessed by MYC staining on T cells from four individual healthy donors. **(b)** MFI of MYC staining representing surface CAR expression levels among transduced T cells. **(c)** $CD4^+$ and $CD8^+$ T cell composition within CAR-expressing populations.
